# Introductory

**Published:** 1907-04-13

**Authors:** 


### Resident Medical Officers' Department.

### INTRODUCTORY.

The point of view of the resident medical officers to our hospitals is one which is not usually considered in the medical press. There is a tendency to regard those who hold responsible house appointments as being still *in statu pupillari*, and their opinions as being prejudiced and immature. The interests and welfare of this section of medical men do not receive at the hands of professional journals so much attention as they deserve.

In nearly every instance of conflict between general practitioner and hospital resident, the bias of medical journalism has been strongly in favour of the former. But we are convinced that this attitude is a wrong one, and prejudicial to the best interests of the profession. The point of view of the hospital resident can not only be made interesting and instructive to his seniors in general practice, but it is one which, if properly represented, will be of service in solving some of the problems which are continually before us. As an example may be mentioned the problem of the abuse of charity and the excessive free relief at our hospitals; this is dealt with in the following article.

In organising a resident medical officers' section in this paper we believe that we shall be doing a service to the profession. The interests of the house surgeon and of the general practitioner are really one; the gap between them is more artificial than real, and it will be the aim of this column to bring into closer touch these two branches of medical practice. Practitioners who have held hospital appointments in the past may find something of interest and of profit in the affairs and opinions of those who now fill the posts which they themselves formerly filled. Furthermore, many of these young men are soon to become their assistants, their partners, or, it may be, their professional opponents. The mental attitude and the characteristics of the present generation of hospital residents are, therefore, worthy of sympathetic attention.

Finally, this section should appeal strongly to those who are now holding hospital appointments. It is intended to represent their interests, to open to discussion the problems of the hospital from within, and to afford a means of communication between the various members of their own branch of the profession. The work of the house physician and the house surgeon is naturally, and rightly, of absorbing interest to themselves. They will find in this column articles on the subjects which intimately concern them, written by those who are not only in sympathy with their point of view, but are also in immediate touch with their own work.

Combined action between resident medical officers throughout the kingdom will strengthen their position and widen their outlook; while co-operation between resident medical officers and the great body of general practitioners will help to remedy many of the abuses which now mock the progress of medicine.

